# Study on Mechanical Failure Behavior of Steel-Wire Wound Reinforced Thermoplastic Pipe under Combined Internal Pressure and Soil Landslide Conditions

**DOI:** 10.3390/ma16020848

**Published:** 2023-01-15

**Authors:** Jun Shi, Zhijie Hu, Li Zeng, Panlin Lu, Hanxin Chen, Nanming Yu, Xiang Li

**Affiliations:** 1 Hubei Provincial Key Laboratory of Chemical Equipment Intensification and Intrinsic Safety, School of Mechanical and Electrical Engineering, Wuhan Institute of Technology, Wuhan 430074, China; 2 Hubei Provincial Engineering Technology Research Center of Green Chemical Equipment, School of Mechanical and Electrical Engineering, Wuhan Institute of Technology, Wuhan 430205, China; 3 Department of Pressure Vessel, China Special Equipment Inspection & Research Institute, Beijing 100029, China

**Keywords:** reinforced thermoplastic pipe, structural failure, finite element analysis, landslide, internal pressure

## Abstract

A steel-wire wound reinforced thermoplastic pipe (SWW-RTP) has been widely utilized in many industrial areas, and a soil landslide is an inevitable hazardous extreme condition for the SWW-RTP as it is usually buried underground. It is imperative to study the mechanical failure behavior and the failure criterion of the SWW-RTP under the combination of internal pressure and soil landslide conditions, and this paper is the first study to investigate the topic. In this paper, groups of stress–strain curves of high-density polyethylene (HDPE) and steel wires were obtained by uniaxial tensile tests at different strain rates, with the help of a Digital Image Correlation device (DIC). A rate-dependent constitutive model was employed to represent the mechanical behavior of the HDPE and to help deduce the stress–strain curve of the HDPE under the required strain rate, estimated from the static simplification of the dynamic soil landslide. Afterwards, a finite element model of the SWW-RTP, embedded in a cubic of soil, was established with the software ABAQUS. The SWW-RTP model was composed of HDPE solid elements, embedded with steel-wire truss elements, and the soil was characterized with the extended Drucker–Prager model. A quartic polynomial displacement distribution was applied to the soil model to represent the soil landslide. Then, the mechanical response of the SWW-RTP was analyzed. It was found that the failure criterion of the HDPE yield was more suitable for the pipe subjected to internal pressure and soil landslide conditions, instead of the steel-wire strength failure criterion always used in traditional research on the SWW-RTP. Further, the influence of landslide width, internal pressure and steel-wire number were discussed. The larger the width of the landslide area, the gentler the deformation of the pipeline; this resulted in an increase in the maximum landslide and a decrease in the maximum curvature with the width of the landslide area. The relatively high internal pressure was beneficial to the safety of the SWW-RTP under landslide, because the internal pressure could increase the stiffness of the pipeline. The number of steel wires had a limited influence on the maximum landslide required for the SWW-RTP’s failure. This work can be useful for the design and safe assessment of the SWW-RTP under internal pressure and soil landslide conditions.

## 1. Introduction

**Figure 1:**
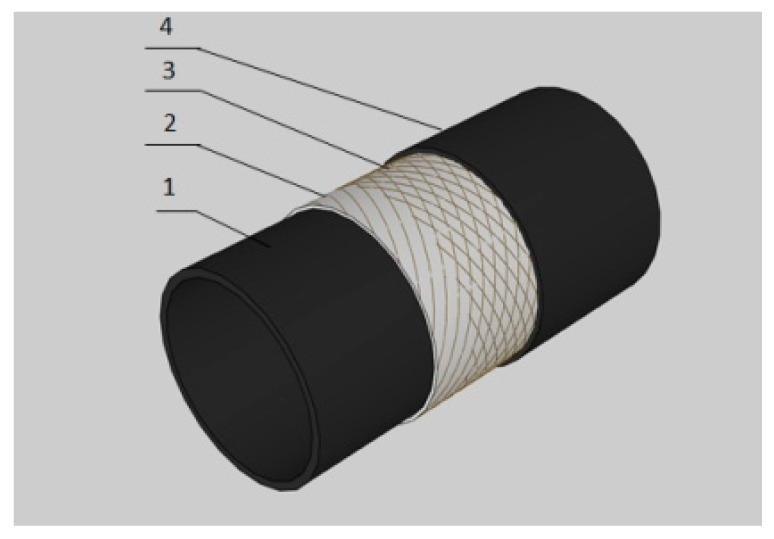
1-HDPE core pipe; 2-inner steel wires; 3-outer steel wires; 4-HDPE cladding layer. Schematic drawing of the SWW-RTP [3].

A steel-wire wound reinforced thermoplastic pipe (SWW-RTP) is a new type of composite pipe that originated in China over ten years ago. The SWW-RTP is composed of high-density polyethylene (HDPE), high-strength steel wires and hot-melt adhesive resin, as shown in Figure 1. The HDPE is used as a core pipe in contact with the medium, and steel wires are cross helically wound around the HDPE pipe to undertake the majority of the load, such as of internal pressure. The hot-melt adhesive resin combines the HDPE and the steel wires together to form the integral structure of the SWW-RTP. Due its unique structure, the SWW-RTP usually possesses a thin wall to undertake the relatively high internal pressure. Relatively high strength, excellent corrosion resistance, etc. [1] make the SWW-RTP widely utilized in many industrial areas, such as the petroleum, chemical, and water supply industries; thus, its application involves different extreme working conditions. The SWW-RTP is usually buried underground, so the seismic loading, such as with soil landslides, is inevitable. The occurrence of landslides has caused immeasurable loss and harm to people’s lives and properties in various countries, and landslides may be more frequent for the SWW-RTP of long-distance transportation that is buried in the remote and mountainous areas [2]. It is imperative to study the mechanical failure behavior of the SWW-RTP under a combination of internal pressure and soil landslide conditions. In addition, it is important to determine the failure criterion of the SWW-RTP in the corresponding service condition for the safety assessment of engineering applications involving soil landslides.

There have been many investigations into the mechanical behavior of the SWW-RTP under different loadings. Jinyang Zheng and Xiang Li et al. [4,5] developed a four-layer analytical model to investigate the mechanical properties of the SWW-RTP under internal pressure, and the four layers included an inner HDPE layer, an inner steel-wire layer, an outer steel-wire layer, and an outer HDPE layer. The model considered the torsion caused by the differences between the winding angles of the inner and outer steel wire layers, based on the structural mechanics. To acquire the elastic parameters of each layer, a meso-mechanical model was used, considering a representative volume element of steel-wire layers. Through the model, the stresses and strains of the four layers and pressures between the interfaces were obtained. Because the steel wires undertook the majority of the load, and their flexibility was much lower than that of the HDPE, the breakage of the steel wires was considered as the failure criterion of the SWW-RTP. Therefore, a strength failure criterion for the SWW-RTP was proposed, taking account of the maximum steel-wire stress, and the failure criterion was validated; indeed, a good agreement between the theoretical results and the experimental data was observed with the relative error only ranging from −5.0% to 4.1% between the calculation and the test results. Jinyang Zheng and Jun Shi et al. [6] carried out theoretical analyses and experimental investigations to understand the relationship between the burst pressure of the SWW-RTP and the varying environmental temperature. Based on the classical laminated-plate theory, a theoretical calculation method was proposed to predict the burst pressure of the SWW-RTP with the SWW-RTP failure criterion, considering the strength failure of the steel wires. Groups of HDPE uniaxial tensile tests were conducted under various temperatures to obtain different elastic HDPE moduli at different temperatures, which were substituted into the analysis model to represent the variation in the SWW-RTP’s properties. Short-term SWW-RTP burst tests were conducted under various temperatures to validate the proposed method. The theoretical results agreed well with the test results, with relative error ranging from 6.72% to 8.82%. Jun Shi et al. [7] constructed a new finite element model based on the actual steel wire spiral structure of the SWW-RTP, in order to consider the nonlinear properties of the steel wires and the HDPE matrix. This work overcame the problem that meant the traditional theoretical analysis model could only take account of the SWW-RTP’s elastic mechanical behavior. In the new finite element model, the steel wires and the HDPE matrix were modeled separately and were represented by solid elements. The interaction between the steel wires and the HDPE matrix was characterized by a tie constraint. The experimental results for the short-term burst pressure of the SWW-RTP was used to validate the nonlinear model. The calculation results of the nonlinear model agreed well with the experimental result with a relative error of −11.38% while the relative error was −37.89% for the linear elastic model. The failure criterion of the SWW-RTP model was still based on the strength failure criterion of the steel wires. Jianfeng Shi et al. [1] investigated the bearing capacity of the SWW-RTP under combined internal pressure and the bending moment at various temperatures. A mechanical testing system was specifically designed to conduct the bending test for the SWW-RTP, performed with a pre-set internal pressure at temperatures varying from 20 °C to 60 °C. A finite element model was subsequently established to analyze the failure process and influence factors of the SWW-RTP under combined loads. The failure criterion of the SWW-RTPs was again verified in the paper, considering steel-wire maximum stress. The relative error of the limit-bending moments at different temperatures was between −3.09% and 6.56%, between the numerical and test results.

In the aforementioned research, the failure criterion of the SWW-RTP was equivalent to the maximum stress criterion of steel wires, which was validated by theoretical and experimental investigations regarding the SWW-RTP in a relatively small deformation. As for the SWW-RTP undergoing a relatively large deformation, due to the landslide and internal pressure, the failure criterion needed to be explored.

Research on the SWW-RTP under landslide conditions was rarely reported, but many researchers have conducted investigations into the mechanical behavior of other types of pipes under landslide conditions. Xiangpeng Luo [8] developed a finite element model for polyethylene (PE) pipes, subject to a seismic landslide. In this paper, the deformation of the PE pipe, due to a seismic landslide, was calculated, and a failure criterion for the pipe was proposed, involving PE yielding. In the proposed finite element model, a quartic polynomial bending deflection displacement, perpendicular to the pipeline, was applied along the axial direction of the PE pipe. The numerical simulation results revealed that the main failure mode of the buried PE pipe, subjected to a seismic landslide, shifted from bending deformation to ovalization deformation with an increase in the bending deflection. Based on the deformation analysis, failure criterion curves were proposed, in order to demonstrate the maximum relative deflection of the pipe cross-section, and the maximum displacement of the pipe versus pipe length subjected to a seismic landslide. Liao Y, etc. [9] carried out investigations on the dynamic response of a gas pipeline horizontal to the landslide. This paper clarified that a relatively large deformation would occur when the pipeline was faced with a landslide, and the deformation of the pipeline increased with the rise displacement caused by the landslide. Owing to their own weight, the middle section of the pipeline was the most dangerous part under the influence of the landslide load. Farzad Talebi and Junji Kiyono [10] used validated 3D solid finite element (FE) models to acquire an accurate sense of the performance of buried pipelines at earthquake faults. The authors improved the modeling techniques in the beam–spring FE modeling approach for the reproduction of the realistic performance of buried pipelines, and determined an appropriate damage criterion for buried pipelines in beam–spring FE models. After the verification of FE models by pull-out and lateral sliding tests, the buried pipeline performance was evaluated at a strike-slip fault crossing, using nonlinear beam–spring FE models and nonlinear 3D solid FE models. Material nonlinearity, contact nonlinearity, and geometrical nonlinearity effects were considered in all analyses.

The researchers mentioned above have carried out systematic studies on different buried pipes, which provide good guidance in the following related research. However, the SWW-RTP has a different structure and failure modes from those pipes in the mentioned papers, so it is necessary to study the failure behavior of buried SWW-RTPs under the combination of internal pressure and soil landslide conditions.

In this paper, a finite element model for the SWW-RTP was established, considering the combined internal pressure and soil landslide conditions, and was used to figure out the SWW-RTP’s mechanical failure behavior and the suitable failure criterion. In the first step, groups of uniaxial tensile tests for the HDPE and steel wires were conducted with the help of a Digital Image Correlation device (DIC). As for the HDPE of typical viscoelasticity, different strain rates were required during the tests to provide necessary test data for acquiring the constitutive equations of the HDPE. Secondly, a finite element model of the SWW-RTP, embedded in a cubic of soil, was established with the software ABAQUS. The HDPE matrix of the SWW-RTP was represented by solid elements, and the steel wires were simulated by truss elements embedded in the HDPE elements. The adhesive interaction between the steel wires and the HDPE was assumed perfect in this paper and the adhesive resin was omitted. The nonlinear properties of the constituents were input into the model. The soil was characterized with the extended Drucker–Prager model. A finite sliding contact was established to represent the soil–SWW-RTP interaction. Then, a quartic polynomial displacement distribution was applied to the soil model to represent the landslide. In the end, based on the model, the mechanical response of the SWW-RTP was obtained and the influence of different factors on the SWW-RTP failure was discussed. In the past research, the steel-wire strength failure criterion was employed for the SWW-RTP, considering internal pressure, and the criterion was validated by different experimental investigations. However, as for the combination of soil landslide and internal pressure conditions, a new failure criterion for HDPE yielding may be more suitable, based on the simulation results. Furthermore, the influence of landslide width, buried depth, internal pressure and steel-wire number were discussed. This paper can be useful for the design and safe assessment of the SWW-RTP under internal pressure and soil landslide conditions.

## 2. Tested Materials and Work Method

### 2.1. Tested Materials

The HDPE used in the SWW-RTP was produced by Sinopec-SK (Wuhan, China) Petrochemical company limited, with a melting point of 130 °C, melt flow rate of 0.35 g/10 min, density of 0.955 g/cm^3^ and elongation after fracture of more than 800%. As the HDPE is a typical viscoelastic material, its mechanical properties change with different tensile loading rates; therefore, groups of the HDPE tensile tests need to be conducted at different tensile loading rates, to help determine the HDPE property at varying rates, corresponding to the landslide. The steel wire, as the reinforcing material of the SWW-RTP, has the characteristics of high strength, good ductility and so on. The steel wire complies with the requirements of ISO 16650: 2004 Bead wire, and it is the main load-bearing component of the SWW-RTP; in the mechanical analysis of the SWW-RTP, the elastic-plasticity of the steel wire must be fully considered. The steel wire can be considered as independent of the tensile rates at room temperature.

### 2.2. Work Method

#### 2.2.1. Tensile Tests

In this experiment, uniaxial tensile tests for the HDPE and steel wire were performed using the LISHI tensile testing machine (supplied by LISHI INSTRUMENT Co., Ltd. Location Shanghai, China), which could be set for tensile tests at different strain rates. The extensometer used with the testing machine was the SANJ YSJ50 extensometer (supplied by Central Iron & Steel Research Institute. Location Beijing, China), which could measure the uniaxial tensile strain up to 50%. The testing machine was controlled by LISHI Test software during the test. This paper aimed to acquire the rate-dependent constitutive model of the HDPE, so tensile tests were conducted with different strain rates at room temperature. The selected tensile strain rates were $0.005\;s^{-1}$, $0.001\;s^{-1}$, $0.0001\;s^{-1}$, $0.00005\;s^{-1}$, respectively. Before the test, the test parameters were set on the software. The strain was taken as the controlling index of the polyethylene tensile test. After setting the control parameters, the sample was clamped in the fixtures of the tensile testing machine. The LISHI test software could record the data during the test.

**Figure 2:**
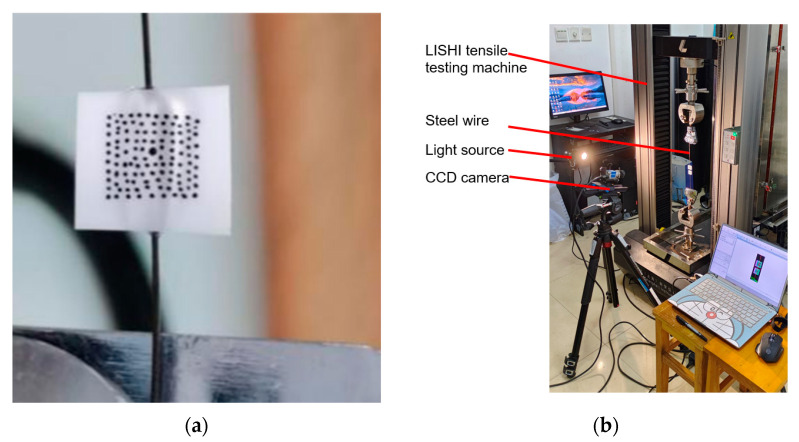
Testing specimens and equipment: (**a**) Speckle image of a single steel wire; (**b**) Testing machine with the 2D DIC system.

Due to the steel wire’s small diameter of 0.6 mm, it is difficult to use the traditional extensometer to measure the strain of the steel wire during tensile tests, so a digital DIC (Digital Image Correlation) [11,12] was employed. Prior to the test, two small pieces of plastic sheet were glued on the steel wire, and a certain distance between the two sheets was reserved as the gauge length for the strain measurement. Speckle images were prepared on the two plastic sheets for the relevant algorithms to identify the change in the relative distance of the two sheets [11,13,14], as is shown in Figure 2a. Then the strain of the steel wire during the test was obtained by dividing the change in the relative distance by the gauge length. The strain measuring equipment, used in the steel wire tensile test, was the VIC-2D test system, developed by the company Correlated Solutions. The VIC-2D test system was mainly composed of hardware (CCD camera, light source, control host) and software (VIC-Snap image acquisition software, VIC-2D V6 Analysis image processing software) [12], as is shown in Figure 2b.

**Figure 3:**
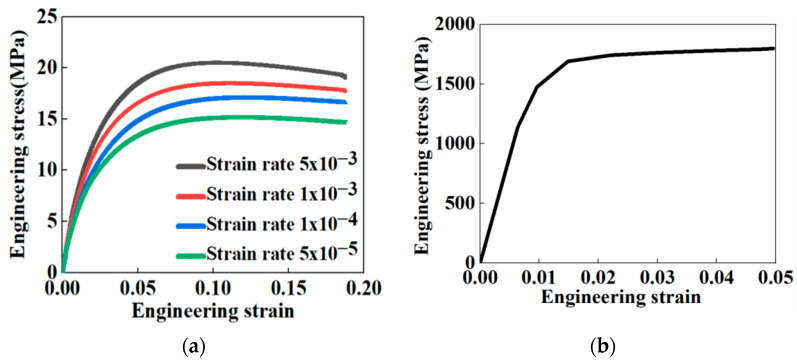
Uniaxial tensile test results: (**a**) HDPE; (**b**) steel wire.

The uniaxial tensile curves of the HDPE and steel wire are shown in Figure 3. As the mechanical properties of the steel wire were independent on the loading rates, the mechanical parameters of the steel wire can be easily obtained from Figure 3. The required material parameters included Young’s modulus of 180,225.21 MPa, a tensile strength of 1845.26 Mpa, and a Poisson’s ratio of 0.26.

Due to its viscoelastic property, studies showed that [8,15] the strength limit and elastic modulus of the HDPE increased with the increase in the strain rate. In the next section, based on the research of Suleiman et al. [16], multiple tensile curves of the HDPE were analyzed under different strain rates and a rate-dependent constitutive model was subsequently proposed [14,17].

#### 2.2.2. Constitutive Model of the HDPE

The velocity of landslide caused by the earthquake was considered between 20 m/s and 70 m/s, and the strain rate of permanent ground deformations caused by the landslide was estimated at about $0.003\;s^{-1}$ [8,18]; therefore, the mechanical property of the HDPE under a strain rate of $0.003\;s^{-1}$ was required in this paper. As is shown in Figure 3, the stress–strain curve of the HDPE demonstrated strong nonlinear properties, so a hyperbolic constitutive model was proposed by deducing model parameters from experiment curves based on Suleiman’ approach [16]. There were two parameters in the constitutive model obtained by curve fitting on the HDPE uniaxial tensile curve. The mentioned stress–strain hyperbolic constitutive relationship is expressed as Equation (1):

(1) $$\sigma =\frac{\varepsilon }{a+b\varepsilon }$$

where, *a* and *b* are parameters related to the tensile strain rate; σ and ε represents the true stress and strain.

Equation (4) can be obtained by transforming Equation (2):

(2) $$\frac{\varepsilon }{\sigma }=a+b\varepsilon$$

**Figure 4:**
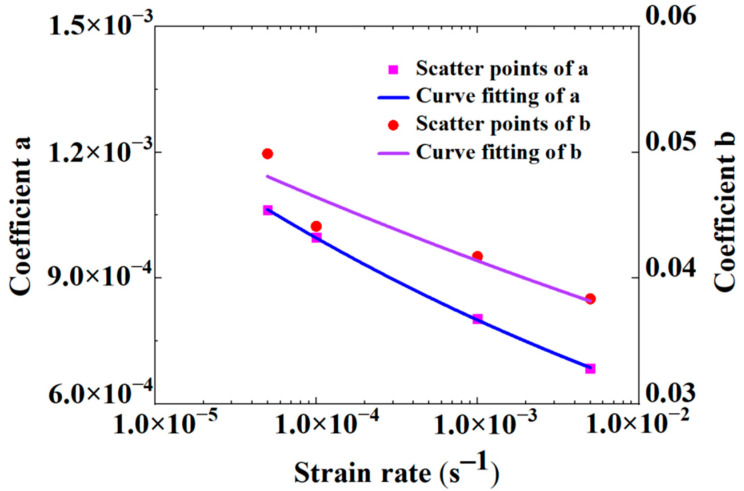
Curve fitting on test results at different strain rates.

**Figure 5:**
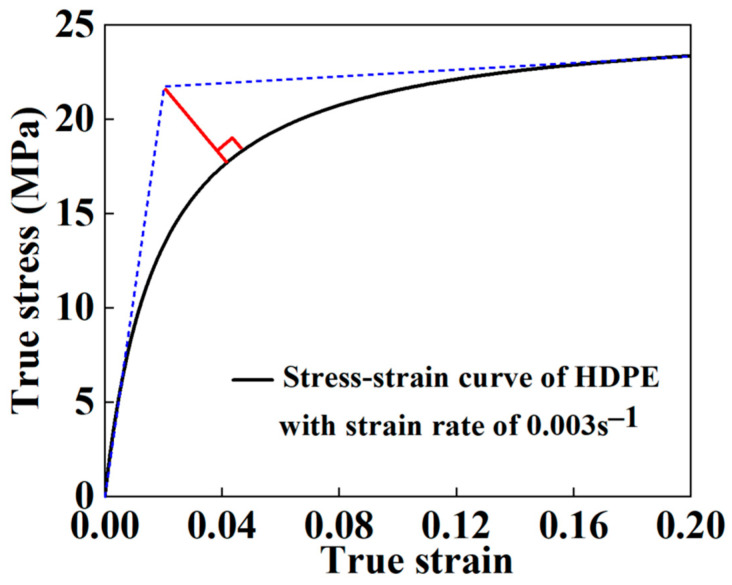
Stress–strain curve of the HDPE at strain rate of 0.003 s^−1^.

Then, the ratio of strain to stress in Equation (2) was taken as the Y value, and the strain was taken as the X value to obtain the values of *a* and *b* by curve fitting, as is shown in Figure 4. The values of *a* and *b* in Equation (2) were listed in Table 1, and the correlation coefficient R, ranging from 0.99745 to 0.99921, indicated that the curve fitting was in very good agreement with the test data. Based on these values, the values of *a* and *b* were expressed as functions of the strain rate, which were listed in Equations (3) and (4), and the parameters in Equations (3) and (4) were listed in Table 2. Therefore, the constitutive equation of the HDPE at any strain rate could be obtained as long as the values of *a* and *b* at the corresponding rates were known. Substituting $\dot{\varepsilon =0.003\;s^{-1}}$ into Equations (3) and (4), the parameters *a* and *b* could be determined, and the stress–strain relationship of the HDPE was found, as shown in Figure 5.

(3) $$a=a_{1}{\left(\dot{\varepsilon }\right)}^{a_{2}}$$

(4) $$b=b_{1}{\left(\dot{\varepsilon }\right)}^{b_{2}}$$

However, for the HDPE of viscoelastic properties, it was difficult to select its yield stress simply from its stress–strain curve, so this paper selected an approximate method to obtain the yield stress of the HDPE [19]. Firstly, two tangential lines were created. One was tangent to the initial part of the curve, and the other was tangent to end of the curve. An intersection point could be obtained with the two tangential lines. Then, a straight line was made along the normal direction of the curve while simultaneously going through the intersection point mentioned in the last step. Thus, another intersection point on the curve could be acquired and the stress corresponding to this point was considered the yielding stress, as shown in Figure 5. The yield strength of the HDPE was 17.74 MPa, and Poisson’s ratio μ was assumed 0.45.

## 3. Finite Element Modelling

### 3.1. Model Structure

The SWW-RTP model was established according to the product of type DN110 PN1.6. The diameter was 110 mm, and the total wall thickness was 8 mm. In total, there were 40 steel wires with a diameter of 0.6 mm. The winding angle was ±54.7.

In the process of a soil landslide, the landslide load is applied to the soil behind the pipeline, pushing the soil and the pipeline to move at the same time. The relevant literature [20,21] proved that the soil model behind the pipeline would have little influence on the mechanical behavior of the pipeline when the width of that part of soil was more than 5 times the diameter of the pipeline. Thus, the corresponding width was set as 0.6 m. The soil in front of the pipeline mainly played a role in preventing the migration, which had a great impact on the mechanical response of the pipeline migration process, and the soil length in front of the pipeline was a significant factor. In order to obtain a simulation result approaching the actual situation, the soil length in front of the pipeline was simulated as infinite by using the infinite elements.

ABAQUS software was used for building the SWW-RTP–soil model. The width of the landslide area was set as 8 m in this paper. To reduce the simulation cost, a 1/2 finite element model for the SWW-RTP and the soil was established. The landslide load and boundary conditions were also symmetrically distributed in the whole model, and the whole soil model was presented as a large cuboid shape.

**Figure 6:**
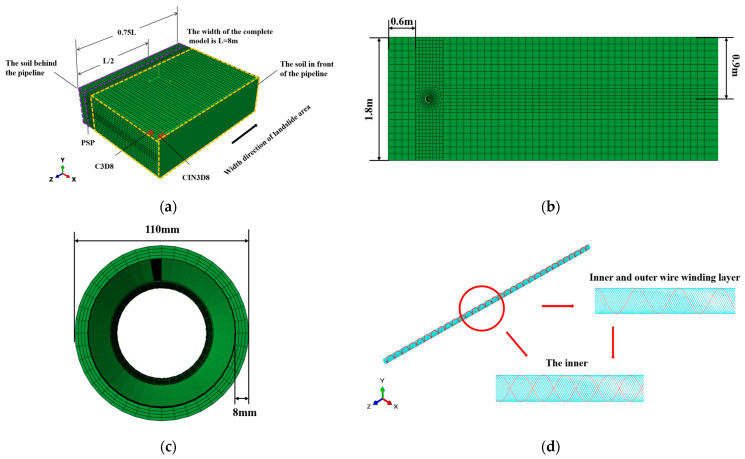
The finite element of the SWW-RTP buried in soil: (**a**) The SWW-RTP–soil model; (**b**) Cross section of soil; (**c**) Cross section of the SWW-RTP; (**d**) steel wires and the HDPE matrix.

The upper surface of the model represented the ground. According to the requirement of Chinese standard [22], the buried depth of the pipeline was set as 0.9 m, as shown in Figure 6a,b. In this paper, N-mm-MPa was used as the default unit when establishing the finite element model with ABAQUS software. L represented the width of the landslide area (Area affected by landslide loading), which meant the length of the affected area was L. The whole length of the SWW-RTP was 1.5 L, and since it was a 1/2 model, only 0.75 L of the pipe was built and L/2 of the soil was applied with a landslide load in the model. The remaining part of the model was a non-landslide area with sufficient width of 0.25 L, as shown in Figure 6a. In the model, the soil was represented by the C3D8 element, and the outermost soil mesh in front of the pipeline was the CIND8 element (infinite element), which was used to simulate the infinite length of soil [23]. The difference between the C3D8 element and CIND8 element is shown in Figure 6a. For the buried SWW-RTP model, the matrix layer and steel wire should be modeled separately, as shown in Figure 6c,d. The embedded interaction module was used to embed the steel wires of truss element T3D2 into the HDPE matrix of the solid element C3D8I. The C3D8I was selected as it could simulate the bending behavior well. This paper focused on the stress and deformation of the pipeline, so it was necessary to refine the soil mesh around the pipeline. Before implementing the numerical calculation, the mesh sensitivity was investigated. Three different kinds of mesh were generated on the pipe and the soil around it, including sparse mesh of 108,424 elements, moderate mesh of 137,752 elements, and refined mesh of 165,872 elements. The Mises stress values of the same bottom point of the pipe were recorded and compared corresponding to the three different meshes, and little difference could be found. In the end, the moderate mesh was selected, and totally, the model had 248,016 elements and 277,601 nodes. Prior to the simulation, the setting of geometric nonlinearity was turned on.

In this paper, the extended Drucker–Prager model was selected to describe the behavior of the soil. The soil was clay and isotropic hardening. The material parameters of the soil were set with reference to publications [8,24] and are shown in Table 3 and Table 4.

### 3.2. Boundary Conditions

**Figure 7:**
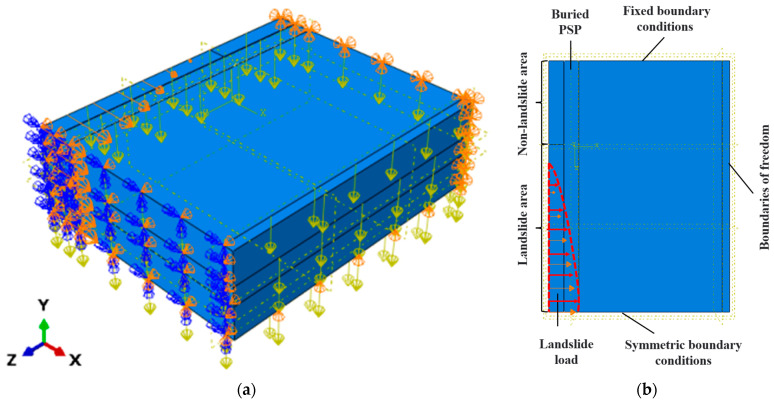
Boundary conditions of the finite element model: (**a**) Isometric view; (**b**) Top view.

As shown in Figure 7a,b, in the finite element model established in this paper, the boundary conditions of soil were as follows: displacement constraint was imposed at the farthest end of the non-landslide area ($U_{X}=U_{Y}=U_{Z}=0$); symmetry constraints were imposed on the symmetry plane at the end of the landslide area; and Y-direction displacement constraints were imposed on the bottom of the whole model [8]. When setting the contact behavior between the pipe and soil, the normal behavior was set as “hard contact”. Its tangential behavior was simulated by the Coulomb friction model, and the friction coefficient could be defined by choosing “penalty” formula in the software.

An internal pressure of 0.6 Mpa was applied to the inner wall of the pipeline, and gravity was applied to the whole model considering the dead weight of the pipe and soil system [8].

According to a field investigation data in the publication [8,23,24], the landslide load could be described in the form of displacement field expressed by a quartic polynomial, shown in Equation (5).

(5) $$X=m{\left(z-\frac{L}{2}\right)}^{4}+n{\left(z-\frac{L}{2}\right)}^{2}+c$$

where, x, z are coordinate values on the X axis and Z axis, respectively, mm; *m*, *n* were the parameters to be determined; *c* was the assumed value of the maximum offset, mm; L was the pipe length in the landslide area, mm. The unknown parameters $m=2.2\times 10^{-12}$ and $n=-1.1\times 10^{-4}$ in this paper were obtained by calculation with a peak displacement of 556.6 mm.

### 3.3. Failure Criterion

In the SWW-RTP, the deformations of the HDPE and the steel wires would coordinate with each other. As the elongation, after the breakage of the steel wires, was much lower than that of the HDPE, the steel wires always broke prior to the strength failure of the HDPE. Thus, it was reasonable to apply the maximum stress criterion of the steel wires, which meant that the SWW-RTP was considered to fail as soon as the stress of the steel wire exceeded the strength limit. This was validated by many burst tests for the SWW-RTP, undertaking only internal pressure [4,5,6,7].

However, as for the SWW-RTP under the combination of internal pressure and soil landslide conditions, the deformation was quite large, and some sections were distorted; meanwhile, the stress of the steel wires did not reach its ultimate strength. This indicated that the structural failure occurred instead of the strength failure. Therefore, the maximum stress criterion of the steel wires was not sufficient to assure the safety of the SWW-RTP for the combination of internal pressure and soil landslide conditions. In this paper, the yield of the HDPE would be considered as the failure criterion of the SWW-RTP firstly, and in the discussion of the influence of different factors on the mechanical behavior of the SWW-RTP, the simulation would continue until the stress of the steel wires approached its ultimate strength. The mechanical response of the SWW-RTP corresponding to the two different failure criterions would be compared, and then the suitable failure criterion would finally be obtained.

The yield of the HDPE meant that the Von Mises stress of the HDPE reached its yielding strength. The definition of the Von Mises equivalent stress is shown as Equation (6).

(6) $$\sigma_{y}=\sigma_{eq4}=\sqrt{\frac{1}{2}\left[{\left(\sigma_{1}-\sigma_{2}\right)}^{2}+{\left(\sigma_{3}-\sigma_{2}\right)}^{2}+{\left(\sigma_{3}-\sigma_{1}\right)}^{2}\right]}$$

where $\sigma_{y}$ is the yield strength of polyethylene (in Mpa) and $\sigma_{eq4}$ is the Mises equivalent stress (in Mpa).

## 4. Results and Discussions

### 4.1. Stress Analysis of the SWW-RTP

**Figure 8:**
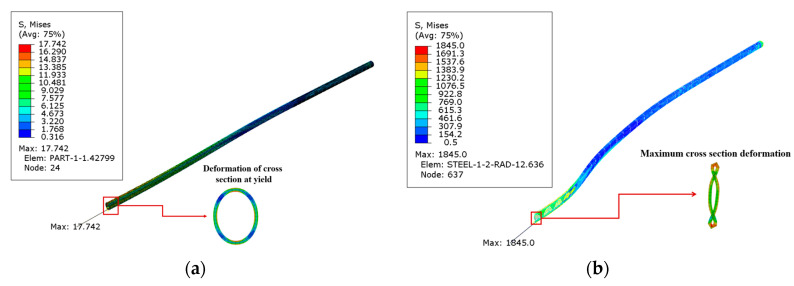
Mises stress distribution of polyethylene and steel wires in the SWW-RTP: (**a**) Mises stress distribution of the SWW-RTP when the HDPE begins to yield; (**b**) Mises stress distribution of the SWW-RTP when the steel wire’s ultimate strength is reached.

Figure 8a shows the Mises stress distribution of the SWW-RTP when the HDPE begins to yield under the landslide of 322.67 mm. Figure 8b demonstrates a more serious distortion of steel wires when the steel wires’ ultimate strength is reached. The change in the cross-section of the SWW-RTP in Figure 8a indicates that the transportation capacity of the SWW-RTP is affected to some extent, while in Figure 8b, the serious distortion results in a sharp drop in the pipe transmission capacity. This comparison implies that the occupation of the HDPE yield as the failure criterion of the SWW-RTP is more suitable than the traditional failure criterion of the steel wires’ breakage.

**Figure 9:**
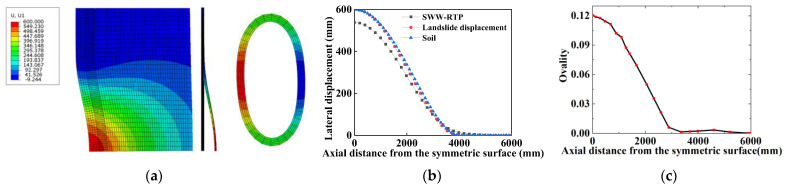
The deformation of the SWW-RTP in soil. (**a**) contour plot of deflection of the SWW-RTP; (**b**) SWW-RTP deflection distribution; (**c**) flattening deformation of cross-section along the pipe’s axial direction.

Figure 9a shows the two main deformations of the SWW-RTP, including the axial bending of the pipe and the flattening deformation of the cross-section. The axial bending can be represented by the curvature from the deflection of the SWW-RTP shown in Figure 9b, and the deflection of the SWW-RTP is consistent with the boundary condition imposed on the soil. The maximum curvature is found at the symmetrical surface of the model, and the value is 7.88 × 10^−5^, which does not seem large. The axial bending of the SWW-RTP is not serious. The extent of the flattening deformation in the cross-section can be defined by the ovality δ, as is listed in Equation (7). In the model, the maximum δ is also found at the symmetrical surface and its value is approximately 0.12, which can be found in Figure 9c.

(7) $$\delta =\frac{D_{max}-D_{min}}{D_{max}+D_{min}}$$

where δ implies the rate of the cross-section change, $D_{0}$ (unit mm) represents the original external diameter of the pipeline, and $D_{max}$ (unit mm) represents the maximum external diameter of the pipeline after flattening deformation. It can be found that the peak value of the ovality is on the symmetric surface of the pipe, and the ovality declines gradually along the pipe’s axial direction.

**Figure 10:**
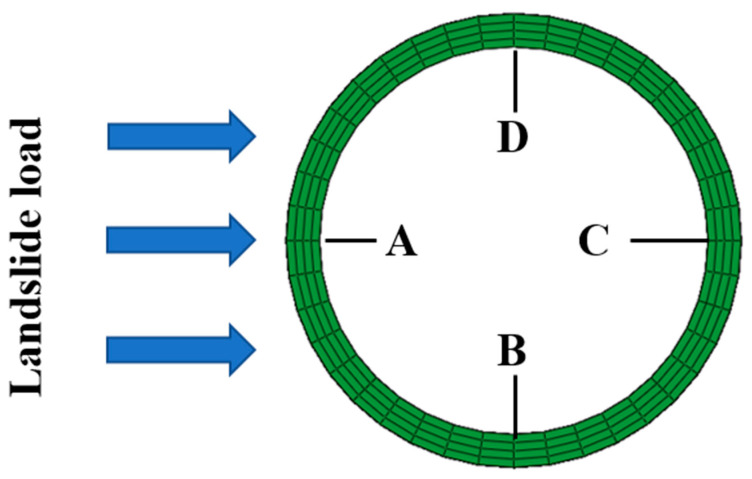
Points A, B, C and D on the symmetric surface of the SWW-RTP model.

**Figure 11:**
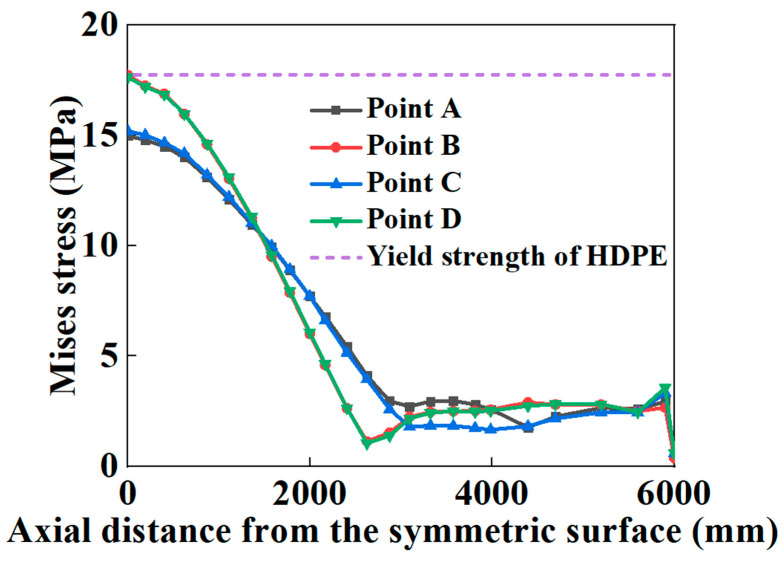
Mises stress distribution of the HDPE matrix along the SWW-RTP axial direction.

When the HDPE begins to yield, the Mises stress at different points of the HDPE matrix, along the pipe’s axial direction, is obtained and depicted. To describe the deformation of the SWW-RTP, four points named as A, B, C, and D are marked in Figure 10. Point A represents the part of the inner surface near the landslide, and point C represents the far part. Point B means the bottom part of the pipe, and the point D means the top part. In Figure 11, it can be seen that the stress of point B and D are higher than that of point A and C near the symmetrical surface of the pipe. This is because the pipe’s bending deformation and the flattening deformation of the cross-section both take effect. The bending deformation leads to the elongation of the pipe and, accordingly, an increase in the tensile stress in the HDPE matrix. The flattening deformation of the cross-section, due to a landslide, results in serious deformation at point B and D; however, there is limited influence on the deformation at point A and point C. When it comes to the section within the coordinate from 3000 to 4000 mm, it can be found that the stress at point A is highest, while the stress at point C is the lowest. This can be explained by the overall deformation shown in Figure 9a. In the section near the coordinate of 4000 mm, the side of the pipe corresponding to point A appears to extend under the effect of the landslide, resulting in tensile stress. However, the side of the pipe corresponding to point C appears to shorten, resulting in compressive stress. Thus, the combination of the tensile effect and internal pressure makes the stress of point A higher than that of point B under the counteraction between the compressive effect and the internal pressure. As for point B and point D within this section, the flattening deformation of the cross-section almost disappears, as shown in Figure 9c. The stress mainly depends on the bending deformation. As point B and point D are on the neutral surface of the pipe, the influence of the bending deformation on these two points is intermediate compared to its influence on point A and point C.

### 4.2. Transformation of Failure Modes

In this study, the exerted landslide distribution is a quartic polynomial, and the deflection distribution of the SWW-RTP caused by the landslide also fits a quartic polynomial. To facilitate the discussion on the landslide, the peak values of the landslide and the corresponding SWW-RTP deflection are, respectively, called landslide and deflection for short; these are used to represent the variation in the landslide loading and the deflection of the SWW-RTP.

**Figure 12:**
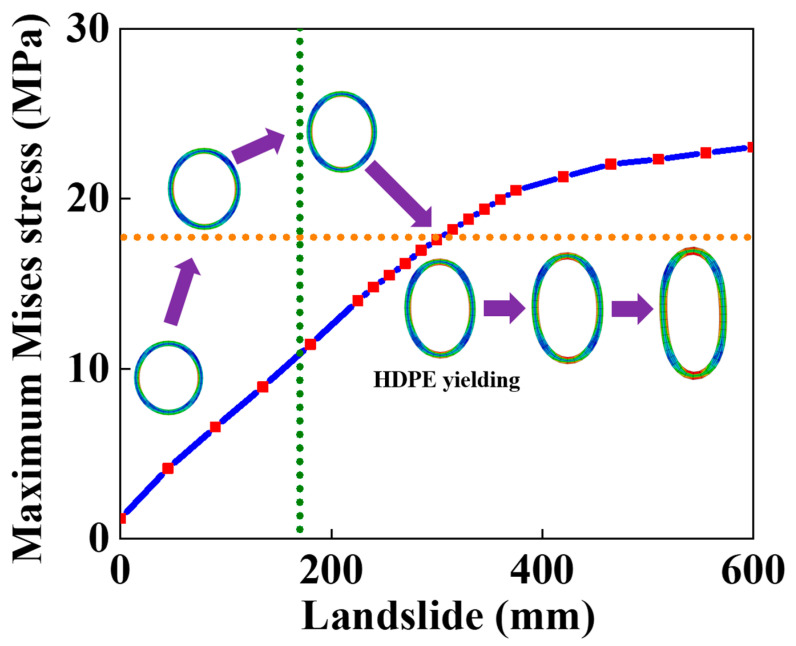
Variation in Maximum Mises stress in the HDPE matrix with the landslide.

**Figure 13:**
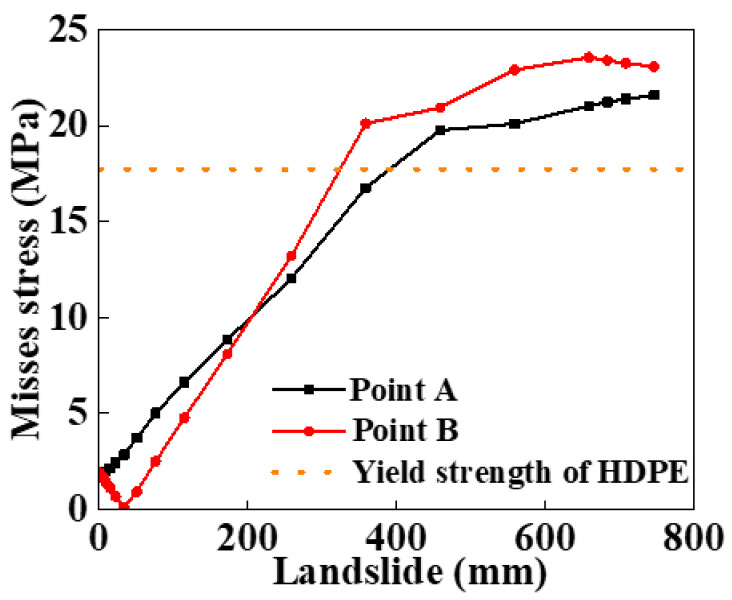

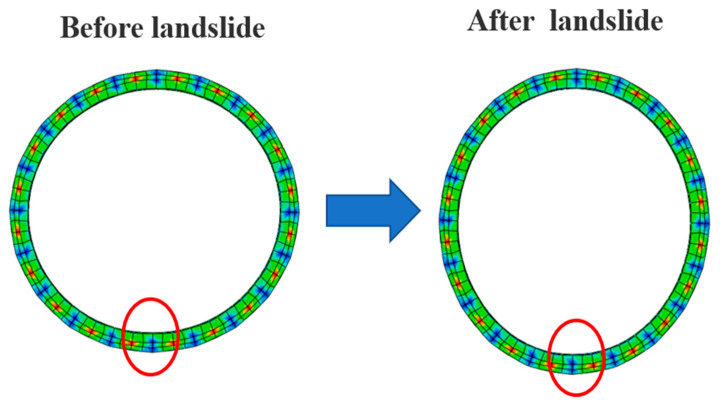
Variation in Mises stress at points A and B on symmetrical surface with the landslide.

The variation in the maximum Von Mises stress with landslide was depicted in Figure 12, when the failure criterion of the yielding of the polymer matrix was occupied. To facilitate the description and the deformation of the SWW-RTP, four points, A, B, C, and D, marked in Figure 10, are still used. Figure 12 indicates that the location of the maximum stress of the HDPE matrix transmits from point A or C to point B or D on the symmetrical surface of the pipe when the landslide increases. The green vertical dotted line represents the transmission of the maximum stress’s location. On the left of the vertical line, the bending of the pipe, along its axial direction, is dominant in increasing stress, so the dangerous points are A or C and the change in the cross-section of pipe is not severe. Conversely, on the right of the vertical line, point B or D becomes the dangerous point, because the squeezing of these two points is the most severe when the pipe begins to be flattened due to large landslide. Figure 13 illustrates the change in the stress at points A and B in more detail. When the peak value of the landslide is lower than approximately 200 mm, the stress of point B is smaller than that of point A. It is noteworthy that the stress of point A rises monotonously, while the stress of point B decreases firstly and then increases. The trend in stress at point A is due to the continuous increasing tensile deformation of the corresponding part in the SWW-RTP. Initially, the internal pressure leads to the tensile strain of point A, and then the landslide pushes that part of point A and makes its tensile strain increase further. Meanwhile, for the bottom part of point B, its curvature decreases, and the tensile strain occurs with the effect of the internal pressure and gravity. When the landslide is applied, the curvature of the bottom part rises and goes back to its original circle shape, so the stress reduces. After the landslide goes up further, the curvature increases further; the stress concentration results in the ascent of the stress, represented by the red curve in Figure 13. Afterwards, when the landslide travels higher, the stress of point B becomes the maximum value of the pipe. This change indicates the transformation of the failure mode. When the landslide is relatively low, the deflection caused by the bending of the pipeline results in the maximum stress of point A; this is because the side with point A is in the tensile state under the effect of bending and the internal pressure. When the landslide becomes higher, the cross-section of the SWW-RTP increasingly deforms, because the soil becomes compact when the landslide increases. The SWW-RTP’s cross-section tends to be flattened. The effect of the flattened trend is more significant, indicated by the maximum stress change in Figure 13; therefore, the transformation of the dominant factor, causing the SWW-RTP failure, gradually occurs as a result of the pipe bending to the excessive deformation of the cross-section.

**Figure 14:**
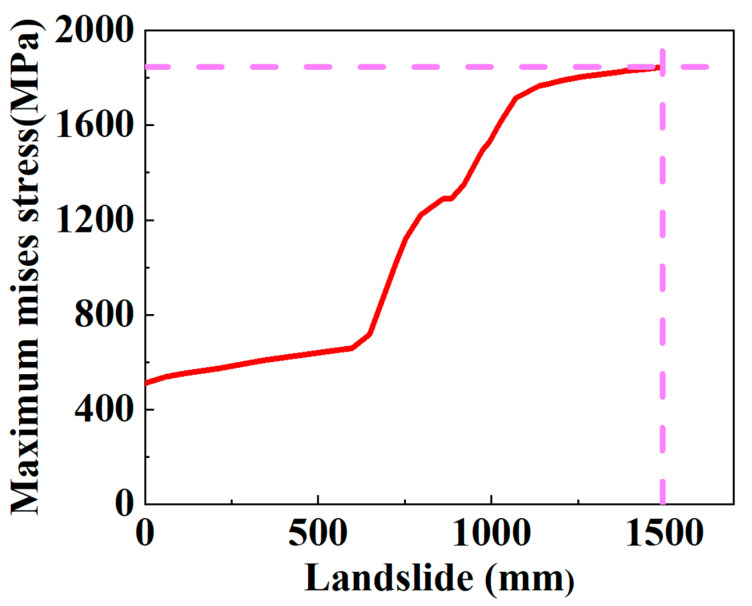
Variation in the maximum Mises stress of steel wires with the landslide.

**Figure 15:**
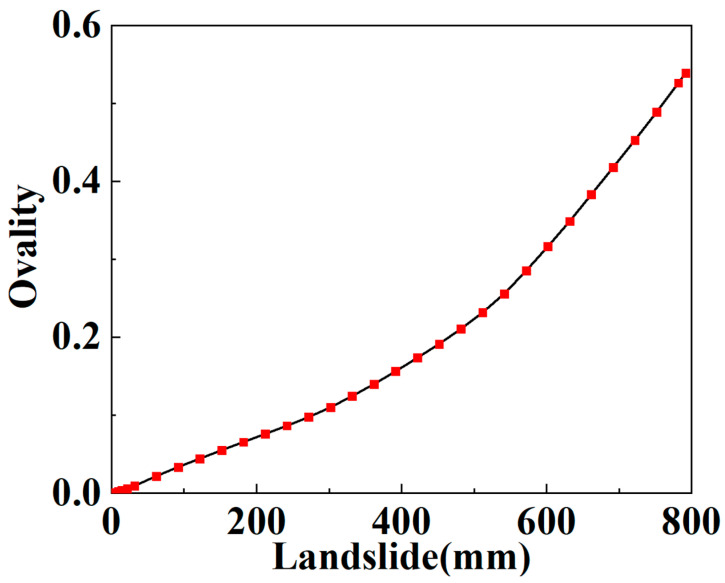
Variation in the maximum δ of the SWW-RTP with the landslide.

When considering the maximum stress criterion of steel wire as the failure criterion of the SWW-RTP, the variation in the stress of the steel wire with the landslide is depicted in Figure 14. The critical landslide, corresponding to the breakage of the steel wire, is about 1500 mm, which is much higher than that corresponding to the HDPE yield at 322.67 mm. However, the SWW-RTP cannot serve normally under that high landslide because of the serious distortion of the pipe’s cross-section. Figure 15 illustrates the variation in the ovality of the SWW-RTP with the landslide. The maximum ovality reaches 0.6 when the landslide is only 800 mm, which is much lower than 1500 mm. Thus, the SWW-RTP works little before the breakage of the steel wires.

## 5. Influencing Factors

The following sections mainly discuss the influence of several important factors on the mechanical failure behavior of the SWW-RTP, including the width of the soil landslide area, the internal pressure, and the number of steel wires.

### 5.1. Width of Landslide Area

**Figure 16:**
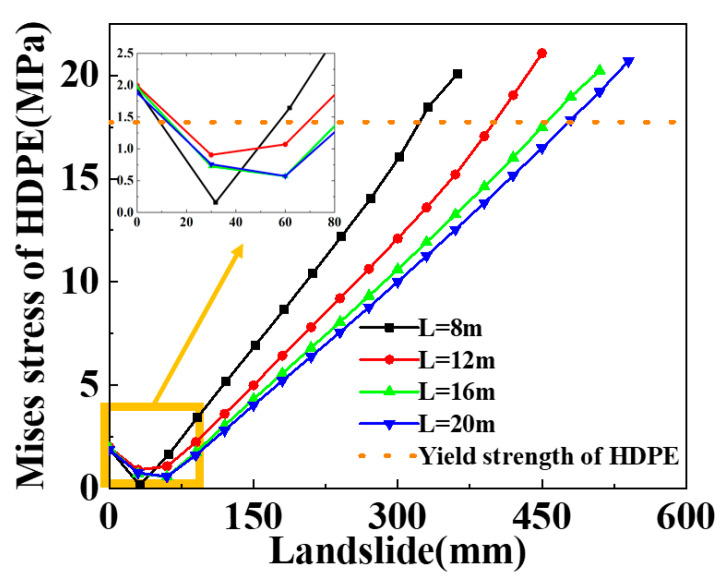
Influence of landslide on the maximum Mises stress of point B with different landslide area widths.

**Figure 17:**
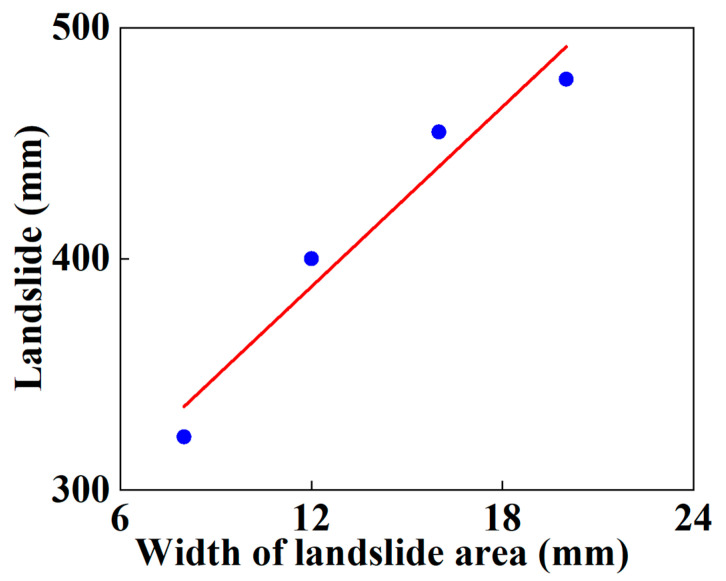
Variation in maximum landslide of the SWW-RTP with different soil widths.

Different landslide width values of 8 m, 12 m, 16 m and 20 m are selected to figure out their influence on the mechanical response of the SWW-RTP. It is shown in Figure 16 that the variation in the stress at point B on the symmetrical surface corresponds to different landslide widths when the landslide increases. It can be found that different curves have a similar ascending trend. Before the landslide is applied, the stress is already caused by the internal pressure. When the landslide is relatively high, the stress rises monotonously. However, when the landslide is lower than 80 mm, the stress at point B first reduced and then increases with the increase in the landslide. This trend is similar to the red curve in Figure 13. This trend is due to the change in the elliptical cross-section, caused by the landslide. During the process of the ellipse’s change, the strain at the bottom point B is, at first, relieved and then increased; therefore, the stress firstly decreases and then increases at the bottom point B. When the stresses of each curve, corresponding to the same landslide, are compared, the stress values are inversely proportional to the widths of the landslide area. This is attributed to the stress concentration in the model. The wider the landslide area is, the gentler the deformation of the pipeline is, so the stress level of the symmetrical surface is relatively low with a wider landslide area. The maximum landslide that the SWW-RTP can undertake keeps increasing when the width of the landslide area rises, as is shown in Figure 17. Curve fitting is conducted on the variation in the maximum landslide with landslide area width, and an expression is obtained: Y = 232.30 + 12.98 × X. X means the width of landslide area, and Y means the maximum landslide. The R square of the curve fitting is 0.9478, which indicates that the data fit the expression well.

**Figure 18:**
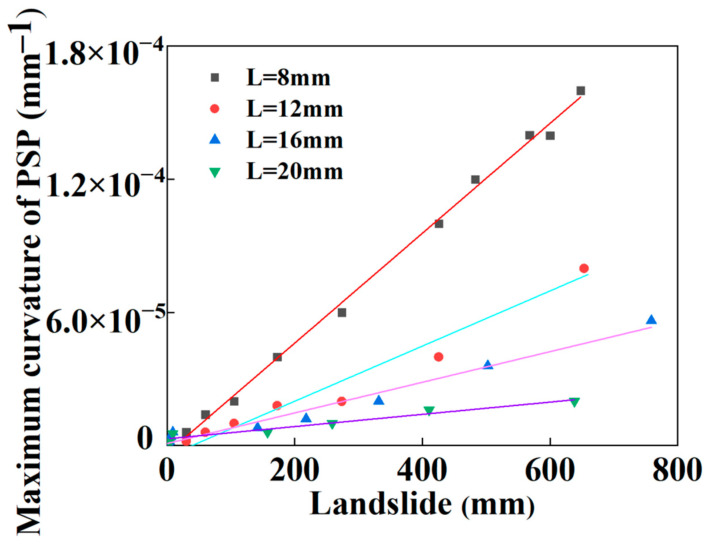
Influence of landslide on the maximum curvature of the SWW-RTP in soil with different landslide area widths.

**Figure 19:**
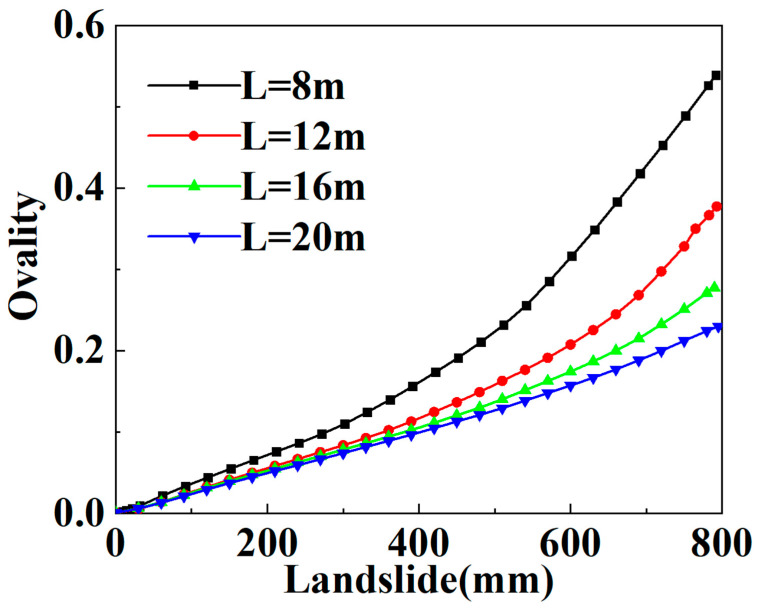
Influence of landslide on δ of the SWW-RTP in soil with different landslide area widths.

**Figure 20:**
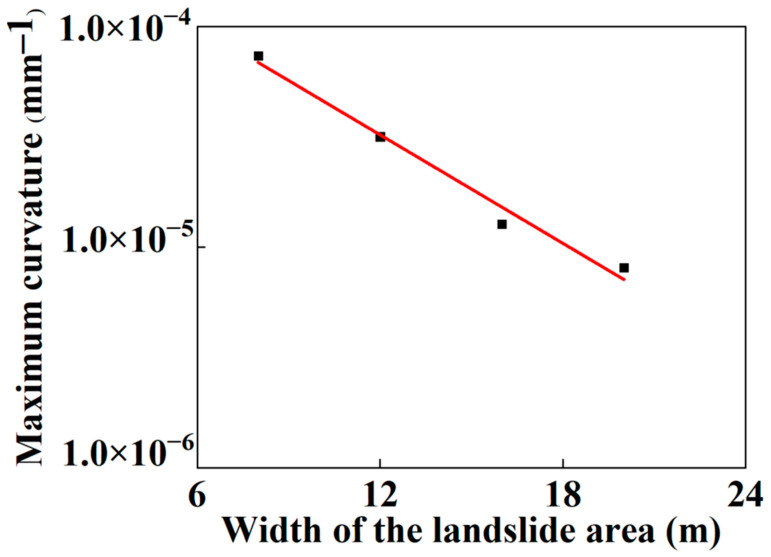
Variation in the maximum curvature of the SWW-RTP with different landslide area widths when the HDPE begins to yield.

**Figure 21:**
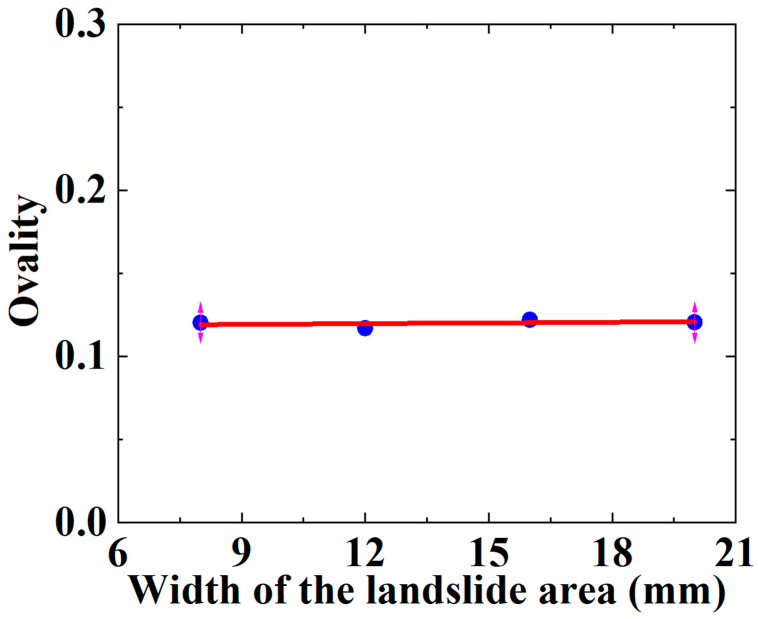
Variation in ovality δ of SWW-RTP with different landslide area widths when the HDPE begins to yield.

As mentioned above, the main possible failure modes of the SWW-RTP pipe deformation are the axial-bending deformation of the SWW-RTP and the flattening deformation of its cross-section. The maximum curvature α is always found on the symmetrical surface of the SWW-RTP, which can represent the extent of the bending deformation, and the ovality δ can characterize the extent of the cross-section’s distortion. In this section, the variations in the α and δ, with different widths of landslide area, are illustrated in Figure 18 and Figure 19, and this can help discriminate the critical factor causing the SWW-RTP failure. As for the SWW-RTP–soil model, with a constant width of landslide area, the increase in the landslide results in an increase in the maximum curvature α and the ovality δ. Meanwhile, the variation in the width of the landslide area leads to different trends in the α and the δ. As is shown in Figure 20 and Figure 21, when the width of the landslide area increases, α declines almost linearly, but δ keeps nearly stable at 0.12. Curve fitting is carried out to find the relationship between the α and the width, which is α = −3.50789 − 0.08195 × X. X indicates the width. The R square of the curve fitting is 0.9823. According to the expression, the maximum curvature α of the pipeline can be determined conveniently instead of via complicated numerical simulations. This can be very helpful for the safety assessment of the pipeline under landslide conditions. Different trends in the α and the δ indicate that the ascending width relieves the extent of the bending deformation, but has little effect on the distortion of the cross-section when the SWW-RTP fails. It can be concluded that the main cause of the SWW-RTP failure is the distortion of the cross-section, instead of the bending of the SWW-RTP, under the effect of the landslide and the internal pressure.

The analysis result might be helpful for the design and safety assessment of the buried SWW-RTP, subjected to the landslide. It is possible to measure the displacement of the pipeline caused by the landslide, the width of the landslide area and the ovality δ. All these parameters can be used to propose a failure criterion for the pipeline with the help of Figure 21. When the measured δ is lower than the stable critical values of those in Figure 21, the pipe can be considered safe. Otherwise, some further analysis needs to conducted to determine the pipe’s safety. The curves in this paper are determined according to a specific type of SWW-RTP, but the analyzing process can also work for the SWW-RTP of other structural parameters.

### 5.2. Internal Pressure

**Figure 22:**
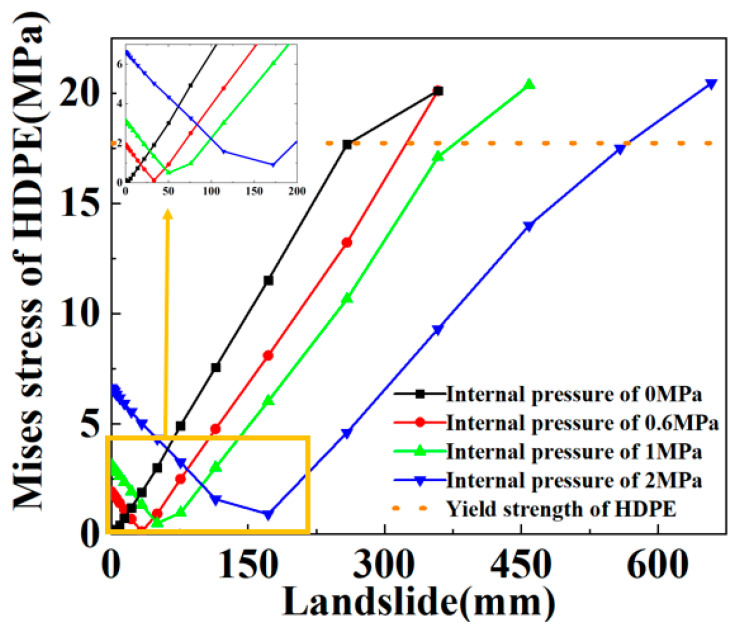
Influence of internal pressure on variation in Mises stress at point B of SWW-RTP with the landslide.

**Figure 23:**
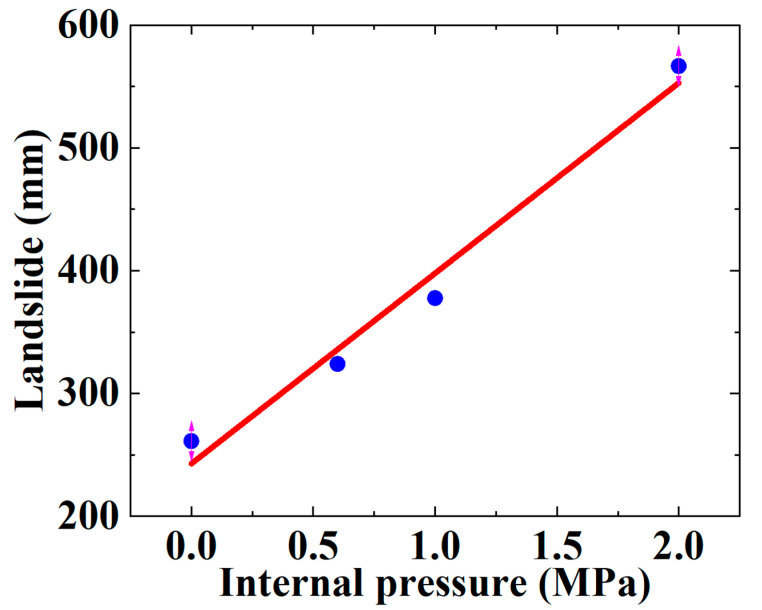
Influence of internal pressure on the maximum landslide of SWW-RTP.

The applied internal pressure in this section is 0 MPa, 0.6 MPa, 1.0 MPa and 2.0 MPa. Figure 22 shows the effect of different internal pressures on the SWW-RTP failure behavior. The stress of the HDPE, corresponding to each internal pressure, first reduces and then rises. This trend is similar to the variation in the stress in Figure 16, and it also results from the competition between the bending deformation and flattening deformation. The maximum Mises stress of the SWW-RTP with a constant internal pressure increases with the rising landslide, which is what is expected. Meanwhile, under the same landslide conditions, it can be found that the maximum Mises stress rises with the decline in the internal pressure; this reflects the internal pressure stiffening the pipe and making the pipe less liable to deform. Figure 23 can be depicted when extracting the maximum landslide corresponding to the failure criterion of HDPE yielding from Figure 22. Figure 23 demonstrates that the maximum landslide increases with the ascending internal pressure. Through curve fitting, an expression Y = 242.82 + 155.10 × X is acquired to represent the variation in the maximum landslide with the internal pressure. X indicates the internal pressure, and Y indicates the maximum landslide. The R square of the curve fitting is 0.9794. All of this means that a relatively large internal pressure is beneficial to the safety of the SWW-RTP under landslide conditions. The reason for this is that the internal pressure increases the stiffness of the pipeline. The internal pressure makes it difficult for the SWW-RTP to bend axially and to be flattened. The expression can play an important role in the pipeline design for determining the maximum landslide.

### 5.3. Number of Steel Wires

**Figure 24:**
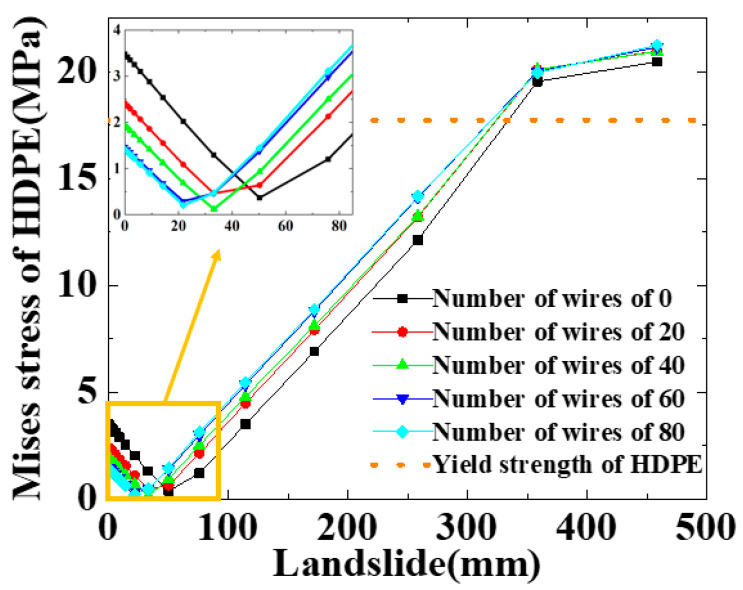
Influence of steel-wire number on variation in Mises stress at point B of SWW-RTP with the landslide.

**Figure 25:**
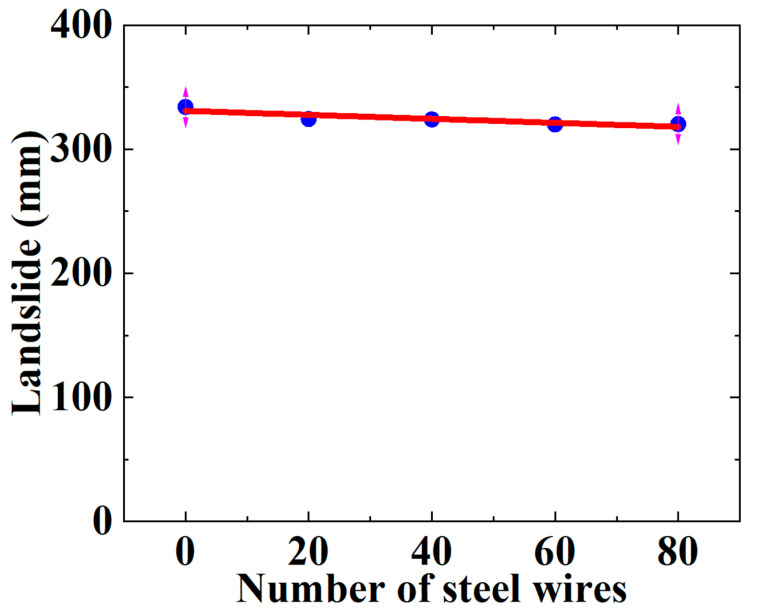
Influence of steel-wire number on the maximum landslide of SWW-RTP.

The number of steel wires plays an important role in the mechanical performance of the SWW-RTP at risk of strength failure. The steel wires are the main load-bearing component of the SWW-RTP, compared with the HDPE matrix. Therefore, this section briefly analyzes the failure behavior of the SWW-RTP under landslide conditions with different numbers of steel wires, including 0, 20, 40, 60, 80; the yielding of the HDPE is considered to be the failure criterion of the SWW-RTP. Figure 24 shows the Mises stress curve of the HDPE matrix in the SWW-RTP with various numbers of steel wires. It can be seen that the number of steel wires has little effect on the yield of the HDPE matrix of the SWW-RTP; this is because the curves possess similar trends and nearly identical critical landslides, corresponding to the yield of the HDPE. This is demonstrated by Figure 25 in detail. The landslide required for the SWW-RTP′s failure is basically the same with different numbers of steel wires. The reason why the number of steel wires does not take effect is that the failure mode of the SWW-RTP is the excessive deformation of its cross-section. The HDPE matrix is mainly responsible for the excessive deformation, and even collapse of the cross-section of the SWW-RTP, because the steel wires can only contain tensile load, and they are too flexible to undertake flexural load.

## 6. Conclusions

A steel-wire wound reinforced thermoplastic pipe (SWW-RTP) is usually buried underground for engineering applications. A soil landslide is an inevitable hazardous extreme condition for the SWW-RTP. However, no publications have previously studied the SWW-RTP under combined internal pressure and soil landslide conditions. This study is the first research to investigate the mechanical failure behavior of the SWW-RTP when subjected to internal pressure and landslide conditions. Groups of uniaxial tensile tests were conducted to obtain the stress–strain curves of the HDPE at different strain rates. A rate-dependent constitutive model was employed to deduce the stress–strain curve of the HDPE under the required strain rate, estimated using the static simplification of a dynamic soil landslide. In addition, the uniaxial tensile curve of the steel wire was obtained using a Digital Image Correlation device (DIC). Then, the nonlinear mechanical properties of the HDPE and steel wires were introduced into a finite element model representing the SWW-RTP buried in soil. A quartic polynomial displacement distribution was applied to the soil model to represent the soil landslide. Accordingly, the quartic deflection distribution of the SWW-RTP was obtained. Based on the model, the following conclusions were made:

1. According to the simulation result, the maximum landslide the pipe can undertake, corresponding to the HDPE yield criterion, is 322.67 mm, while the maximum landslide, corresponding to the breakage of the steel wire, is approximately 1500 mm; however, the failure criterion of the HDPE yield appears more suitable for the loading combination of internal pressure and soil landslide conditions; this is because the cross-section of the pipe becomes elliptical, and the transportation capacity of the SWW-RTP begins to reduce when the HDPE matrix begins to yield. Meanwhile, the SWW-RTP distorts extensively and cannot serve normally, even well before the steel-wire stress approaches its ultimate strength.
2. The SWW-RTP undergoes axial bending deformation and flattening deformation under the soil landslide and internal pressure conditions. Under the effect of the two loadings, the bottom and top points on the symmetrical surface of the SWW-RTP are the dangerous points where the Mises stress values are the highest. When the maximum deflection along the pipe continues to increase, it can be found that the effect of the flattened deformation is more significant than that of bending deformation on the SWW-RTP failure; the dominant factor of the SWW-RTP failure gradually transforms from the axial bending to the flattening deformation of the cross-section.
3. The larger the width of the landslide area is, the gentler the deformation of the pipeline is. This results in the variation in the maximum landslide and the maximum curvature. The maximum landslide rises with the width of the landslide area, and the relationship is that Y = 232.30 + 12.98 × X, where X represents the width, and Y represents the maximum landslide. The maximum curvature α decreases with the width of the landslide area, and the relationship is that α = −3.50789 − 0.08195 × X. X indicates the width. According to the expressions, the maximum landslide and the maximum curvature can be determined conveniently, instead of via complicated numerical simulations, once the width of the landslide area at the scene of the landslide accident can be estimated. This can be very helpful for the safety assessment of the pipeline under landslide conditions.
4. The relatively large internal pressure is beneficial to the safety of the SWW-RTP under landslide conditions, because the internal pressure increases the stiffness of the pipeline, making it difficult for the SWW-RTP to bend axially and to be flattened. An expression Y = 242.82 + 155.10 × X is acquired to represent the variation in the maximum landslide with the internal pressure. X indicates the internal pressure, and Y indicates the maximum landslide. The expression can play an important role in the pipeline design and the related safety assessment.
5. The number of steel wires has a limited influence on the maximum landslide required for the SWW-RTP’s failure, as the failure mode of the SWW-RTP is the excessive flattening deformation of the cross-section. The steel wires only contain tensile load, and they are too flexible to undertake flexural load. In contrast, the HDPE matrix is mainly responsible for the excessive deformation, and even collapse, of the cross-section of the SWW-RTP.

This research will be helpful for the design and safety assessment of the buried SWW-RTP, subjected to soil landslide and internal pressure conditions.

## Figures and Tables

**Table 1:** Fitting parameters of stress–strain curves.

	Strain Rate	5 × 10^−3^	1 × 10^−3^	1 × 10^−4^	5 × 10^−5^
Fitting Parameters					
a		6.8 × 10^−4^	8.0 × 10^−4^	1.0 × 10^−4^	1.1 × 10^−3^
b		3.8 × 10^−2^	4.2 × 10^−2^	4.4 × 10^−2^	5.0 × 10^−2^
R		1.0 × 10^−1^	1.0 × 10^−1^	9.9 × 10^−1^	1.0 × 10^−1^

**Table 2:** Fitting parameters of equations.

a_1_	a_2_	b_1_	b_2_
4.15 × 10^−4^	−9.49 × 10^−2^	2.93 × 10^−2^	−5.01 × 10^−2^

**Table 3:** Parameters of Drucker–Prager model for soil.

ρ (kg/m^3^)	*E* (kPa)	μ	β( ^0^ )	ψ( ^0^ )
1867.3	20,000	0.4	28.7	0

**Table 4:** Hardening parameters of Drucker–Prager model.

$\sigma_{1}-\sigma_{2}\left(kPa\right)$	170.1	649.9	740.3	801.4	848
$\varepsilon_{P}$	0	0.035	0.05	0.073	0.091

## Data Availability

Not applicable.
